# Ultrafast dynamics of photo-excited 2-thiopyridone: Theoretical insights into triplet state population and proton transfer pathways

**DOI:** 10.1063/1.5143228

**Published:** 2020-03-17

**Authors:** Jesper Norell, Michael Odelius, Morgane Vacher

**Affiliations:** 1 Department of Physics, AlbaNova University Center, Stockholm University, SE-106 91 Stockholm, Sweden; 2 Department of Chemistry-Ångström Laboratory, Uppsala University, 75121 Uppsala, Sweden; 3 Laboratoire CEISAM-UMR CNRS 6230, Université de Nantes, 44300 Nantes, France

## Abstract

Ultrafast non-adiabatic dynamics of the small heteroaromatic compound 2-thiopyridone has been studied with surface hopping simulations based on multi-configurational quantum chemistry. Initial excitation of the bright ${\text{S}}_{2}(\pi ,\pi^{*})$ state is found to promptly relax to ${\text{S}}_{1}(n,\;\pi^{*}$) through in-plane motion. The subsequent dynamics are oppositely driven by out-of-plane motion, which results in both complex population transfers among all the available states and intersystem crossing predominantly through the “El-Sayed forbidden” ${\text{S}}_{1}(n,\;\pi^{*}$) to ${\text{T}}_{2}(n,\;\pi^{*}$) channel, through significant mixing of electronic excitation characters. Despite this complexity, the femto- to picosecond triplet population, expected from several spectroscopic measurements, is well described as a simple exponential decay of the singlet state manifold. No proton transfer is found in the reported trajectories, but two mechanisms for its possible mediation in previously reported experiments are proposed based on the observed structural dynamics: (i) ultrafast intra-molecular transfer driven by the initially coherent in-plane motion and (ii) inter-molecular solvent-mediated transfer driven by the out-of-plane modes that dominate the later motion.

## I. INTRODUCTION

Due to the importance of light induced processes in biomolecules, in general, and in nucleic acids, in particular, the photo-chemistry of heteroaromatic compounds has been the subject of extensive investigations.^1^ Among these, thiosubstituted (organosulfuric) compounds have gathered increasing interest from ultrafast science due to their wide range of applications and rich excited-state dynamics.^2–17^ One such compound, 2-thiopyridone (2-TP, see Fig. 2), has primarily been studied as a compact model system for excited-state proton transfer (ESPT),^18–24^ which is related to, for instance, photo-protection mechanisms of DNA^25^ and pigmentation of the human skin.^26^

While it is long established that the nitrogen protonated 2-TP can tautomerize into sulfur protonated 2-mercaptopyridine upon a change in the chemical environment (gas phase, solid, or solvated),^27–31^ it is only recently that time-resolved measurements^18,20–22^ have been used to explicitly investigate the possibility for the corresponding UV-pumped process. In the measurement with the highest temporal resolution, traces of N–H dissociation were found below picosecond (ps) timescales using N1s resonant inelastic x-ray scattering (RIXS), but, due to limited statistics, the electronic states involved in the process could not be resolved.^20^ Two pico- to nanosecond (ns) measurements, performed using N1s^22^ and S1s^21^ near-edge x-ray absorption (NEXAFS), however, instead find the ESPT to be a nanosecond process, consistent with previous vibrational measurements.^18^ In particular, our recent study,^22^ combining time-resolved N1s NEXAFS with spectrum simulations of valence-excited states, provided strong evidence for a dominant reaction pathway on these timescales, shared for S_2_ and S_4_ initial excitation, where ESPT results from nanosecond decay of the T_1_ state. Notably, none of the studies have been able to address whether the ESPT occurs intra-molecularly or inter-molecularly through solvent-mediated formation of a deprotonated anion 2-TP^−^.

The joint body of evidence for the ESPT pathway in 2-TP currently contains several gaps, particularly with respect to the ultrafast dynamics that leads to triplet population. Highly related, as a culmination of many previous experimental^2–9^ and theoretical^10–17^ efforts, the groups of Crespo-Hernández and González recently offered a mechanistic explanation for the general trend of rapid and efficient triplet population in thionated compounds as a stabilization that depends on the localization of the electronic excitation.^32^ The spectroscopic measurements, based on UV absorption, were in this case supplemented with *ab initio* simulations of the non-adiabatic molecular dynamics, where recent extensions of trajectory surface hopping methods^33^ allowed the authors to describe, with relative affordability, non-adiabatic dynamics with spin–orbit induced transitions.^34–36^ With a computational description of the complete photo-chemical process, it can, thereby, be interpreted in well-known chemical concepts such as molecular orbitals, potential energy surfaces, and conical intersections, which may not be possible to similarly extract from measurements.

In the current work, we simulate the non-adiabatic molecular dynamics, including spin–orbit coupling induced transitions, of photo-excited 2-TP. This provides a link between the excited-state dynamics probed in previous time-resolved measurements^18,20–22^ and quantum chemical insights gained from previous simulations.^18,22–24,31^ Our main goal is to identify inherent aspects of the ultrafast dynamics, both electronic and structural, which result in the observed triplet population and may drive ESPT along previously reported or alluded pathways at various timescales.

## II. COMPUTATIONAL DETAILS

Non-adiabatic molecular dynamics of 2-TP was simulated by trajectory surface hopping^33^ as implemented in the SHARC code,^34,35^ version 1.01. The simulations included three singlet (S_0_, S_1_, and S_2_) and two triplet (T_1_ and T_2_) states, as denoted in the basis of molecular Coulomb Hamiltonian (MCH) states (i.e., with pure spin multiplicities as obtained directly from the electronic structure calculations) with propagation performed in the spin-mixed basis of fully diagonal states. A time step of 0.5 femtoseconds (fs) was employed for the nuclear dynamics, with 100 substeps for propagation of the electronic state amplitudes. Non-adiabatic couplings were estimated from the overlap of electronic wave functions at different time steps, referred to as the local diabatic or overlap method.^37–39^ An energy based decoherence correction^40^ of 0.1 Hartree was further applied to correct for the artificially high coherence between different electronic states otherwise expected in trajectory surface hopping frameworks. An ensemble of 100 trajectories was simulated, with initial conditions sampled from a Wigner distribution to reproduce the vibrational ground state of S_0_; all simulations were initialized populating the S_2_ state.

**FIG. 1.**
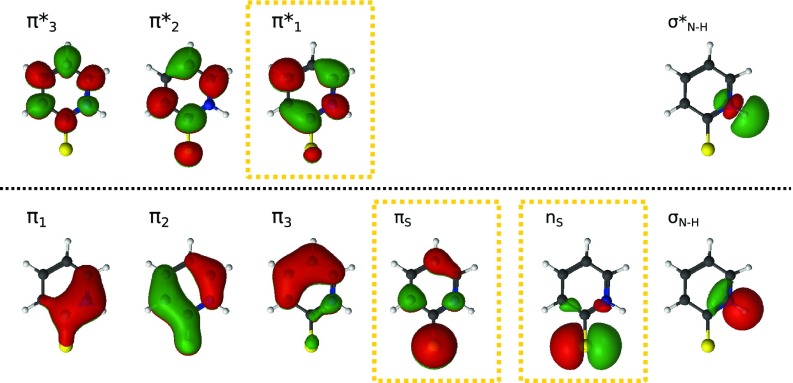
Active molecular orbitals in the CASSCF calculations, here shown for the S_0_ optimized geometry. The studied valence states can nominally be assigned to excitation from the sulfur dominated *π_S_* (S_2_ and T_1_) and the sulfur lone-pair *n_S_* (S_1_ and T_2_) into $\pi_{1}^{*}$.

The underlying electronic structure calculations were performed in OpenMolcas^41^ with a CASSCF(12, 10)^42^ setup that included: the full *π* system ($\pi_{1},\;\;\pi_{2},\;\;\pi_{3},\;\;\pi_{S},\;\;\pi_{1}^{*},\;\;\pi_{2}^{*},\;\;\pi_{3}^{*}$) and the lone-pair $n_{S}$ to describe the valence excitations, together with $\sigma_{N-H}$ and $\sigma_{N-H}^{*}$ to describe the potential breaking of the N–H bond, as visualized in Fig. 1. The same level of theory was also applied to (i) optimize the S_0_ geometry and calculate its vibrational modes and vertical electronic excitations and (ii) optimize pair-wise minimum energy crossing points (MECPs) [or, more specifically, minimum energy conical intersections (MECIs) for states with the same spin multiplicity] between the electronic states. Second-order perturbation theory corrections for the vertical excitation energies in the Franck–Condon geometry were additionally calculated using multi-state CASPT2.^43,44^ Scalar relativistic calculations using the Douglas–Kroll–Hess formulation^45,46^ was performed employing the relativistic ANO-RCC-VDZP^47^ basis set (which is shortly compared to the larger ANO-RCC-VTZP basis in supplementary material Sec. S1) together with Cholesky factorization.^48,49^ Spin–orbit coupling elements were computed using an atomic mean field integral (AMFI)^50^ approach within the RASSI framework.^51,52^ Molecular orbitals were visualized with the luscus program.^53^

Further details of the complete computational setup, in the form of example inputs, are provided in the supplementary material.

## III. RESULTS AND DISCUSSION

### A. Electronic states

**FIG. 2.**
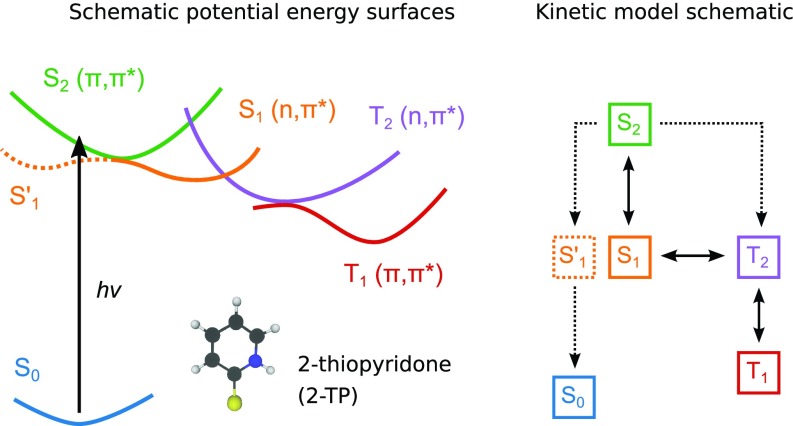
Schematic potential energy surfaces in the region of 2-TP photo-excitation (left). Schematic of the kinetic model derived from the simulations and fitted to the electronic state populations. Solid lines indicate the main pathway and dashed lines the minority pathways (right).

To describe and rationalize the non-adiabatic dynamics, we first introduce the electronic states that will be involved. An overview of the first four excited states of 2-TP and their potential energy surfaces in the region of the explored dynamics, schematically constructed in accordance with our later drawn conclusions, are given in Fig. 2. The electronic excitation energies computed in the Franck–Condon geometry at CASSCF and CASPT2 levels of theory are gathered in Table I(a). The singlet manifold consists of the dark ${\text{S}}_{1}(n,\;\pi^{*})$ and the bright ${\text{S}}_{2}(\pi ,\;\pi^{*})$, whereas the triplet manifold contains ${\text{T}}_{1}(\pi ,\;\pi^{*})$ and ${\text{T}}_{2}(n,\;\pi^{*})$. Including dynamic correlation with perturbation theory leads to energy differences of less than 0.2 eV for singlet states and a bit larger for triplet states. Importantly, the electronic state characters and their energy ordering remain the same upon adding dynamic correlation. It is interesting to remark that the energy ordering of excitation characters ($\left(n,\;\pi^{*}\right)$ and $\left(\pi ,\pi^{*}\right)$) is inverted between the two manifolds (singlet and triplet) and all states are found in a narrow energy range, in particular, the lowest three, at both levels of theory. While the excitation characters may be strictly identified for all states in the currently presented Franck–Condon analysis, we note that the same does not hold through the later presented trajectories. For discussion of the non-adiabatic dynamics, the states will, therefore, in general, simply be denoted by their energy ordered MCH state labels, i.e., without specified excitation character.

**TABLE I.** (a) Excitation energies of the electronic states in the Franck–Condon region. Upper row: directly from CASSCF calculations, as employed for the dynamics. Lower row in parentheses: with added multistate CASPT2 correction. (b) Total number of surface hops from the old MCH state (rows) to a new MCH state (columns) found in the 100 trajectories. Only hops with 10 or more occurrences are shown, and the forward transitions of the main pathway are emphasized in bold.

(a) Excitation energies in units of eV.			
${\text{S}}_{1}(n,\pi^{*})$	${\text{S}}_{2}(\pi ,\pi^{*})$	${\text{T}}_{1}(\pi ,\pi^{*})$	${\text{T}}_{2}(n,\pi^{*})$
3.16	3.82	2.64	2.90
(3.34)	(3.64)	(2.92)	(3.31)

(b) Total number of MCH surface hops.					
From To	S_0_	S_1_	S_2_	T_1_	T_2_
S_0_	…	…	…	…	…
S_1_	13	…	348	…	**298**
S_2_	…	**441**	…	…	10
T_1_	…	…	…	…	4849
T_2_	…	251	…	**4869**	…

In a single-electron picture, all the states nominally result from excitation into the lowest unoccupied molecular orbital (LUMO) $\pi_{1}^{*}$, S_2_ and T_1_ from the sulfur-dominated $\pi_{S}$, S_1_ and T_2_ from the in-plane sulfur lone-pair $n_{S}$. Of particular relevance for later interpretation, we note that $\pi_{S}$ and $n_{S}$ correspond to the nearly degenerate highest occupied molecular orbital (HOMO) and HOMO-1 in a single-determinant description, respectively,^23^ where the sulfur dominance of the former makes it comparable to an out-of-plane lone-pair. Visualizations of all the active molecular orbitals are found in Fig. 1.

### B. Electronic dynamics

Non-adiabatic molecular dynamics simulations were performed to investigate the relaxation upon the population of the S_2_ bright state, with CASSCF as the underlying electronic structure method. A number of studies, based on *ab initio* multiple spawning,^54–63^ in particular, but also on other methods,^17,64^ have previously demonstrated the ability of CASPT2 based simulations of non-adiabatic dynamics to yield even quantitative results. Yet, due to both the difficulty in the implementation of CASPT2 gradients and their steep computational cost, simulations that include spin–orbit coupling have instead mainly been based on the CASSCF level of theory.^65–68^ Due to the lack of dynamic correlation in the current description, later conclusions will primarily be drawn about qualitative rather than quantitative aspects of the simulated dynamics. The simulated trajectories were all started in the bright ${\text{S}}_{2}(\pi ,\pi^{*})$ state, which has previously been experimentally pumped with 343 nm (Ref. 22) and 400 nm (Ref. 21) lasers and shares a common reaction pathway on picosecond timescales and longer with the second bright ${\text{S}}_{4}(\pi ,\pi^{*})$ state,^22^ pumped instead with 258 nm (Ref. 18) and 266 nm (Ref. 22) lasers.

**FIG. 3.**
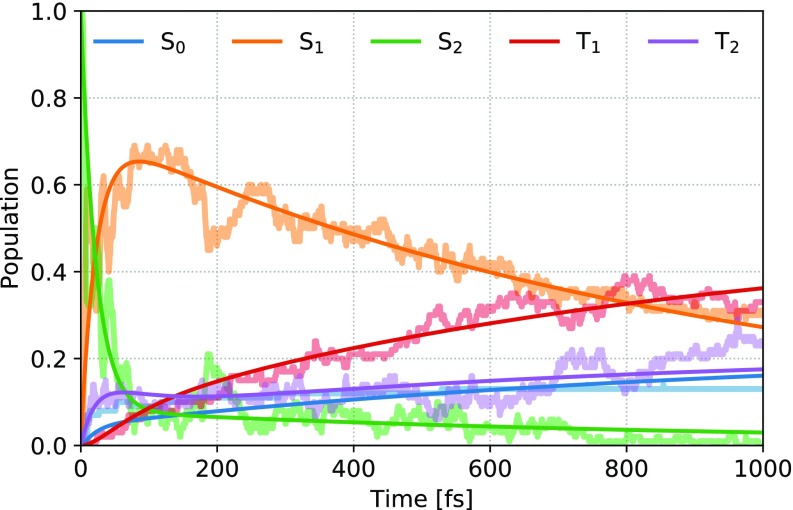
Electronic state populations from the 100 trajectory ensemble (thick shaded lines) compared to the fit obtained with the full kinetic model (thin opaque lines, see supplementary material Sec. S4 for further details).

From the ensemble of trajectories, we first consider the kinetics of the electronic state population transfers. The relative populations of the MCH states as a function of time are shown in Fig. 3 together with a kinetic model fit. Comparison of different state representations (see supplementary material Sec. S3) displays only small differences for the overall evolution of the populations, which thereby demonstrates a stability of the populations themselves despite later discussed complications in the electronic state characters. The main clues to the reaction pathway are found in Table I(b), which enumerates the number of surface hops between the states. The dominant pathway is, in this way, readily identified as a sequential decay in reverse energy order, i.e., S_2_ to S_1_ to T_2_ to T_1_, hereafter referred to as the “main” pathway. Yet, the total kinetics cannot be so easily described for at least two reasons: first, because of the numerous “backward” transitions within the main pathway, which disallows the population transfer to be accurately modeled with a simple exponential “forward” decay and second, due to the existence of several “minority” pathways, which, for instance, partially re-populates S_0_ at very early times.

Our attempts to fit the obtained populations with a kinetic model are described in more detail in the supplementary material, Sec. S4. In short, we find that a model with nine different rate constants (three main forward, three main backward, and three minority forward transitions), as schematically depicted in Fig. 2 and with the resulting fit shown in Fig. 3, is necessary to capture all qualitative aspects of the kinetics. Consequently, the lifetime of individual electronic states can be neither simply nor robustly extracted, as they result from a complex balance between backward and forward transfers of population and are highly sensitive to changes of the fitted model. We assign the complexity in the population kinetics to primarily result from large similarities between the states, both in their excitation energies and excitation characters, as elaborated in this section and Sec. III C.

The small energy splitting of the electronic states, already noted from their vertical excitations, is presumably the main cause for the significant contribution of backward transitions within the main pathway. An estimate of how the energy splittings develop over time, based on the spin–orbit coupled state representation wherein the SHARC dynamics are propagated, is presented in supplementary material Sec. S2. In short, following large fluctuations within the first ∼100 fs, the mean values of the splittings stabilize toward roughly 0.4 eV (S_1_ to T_2_) and 0.3 eV (T_1_ to T_2_). As a result, the S_1_, T_1_, and T_2_ states share comparable populations at 1 ps, in contrast to multiple studies of similar systems where a single T_1_ state dominates at such timescales.^14,17,32^ The increased tendency for backward transitions can also arise from limited “funneling” toward the associated conical intersection in the “upper” state and subsequently away from it in the “lower” state, as previously observed for LiH_2_.^69^ Indeed, comparing the MECIs (which represents well the hopping geometries as shown in Sec. III C) for S_2_–S_1_ and T_2_–T_1_, the former is categorized as peaked and bifurcating and the latter as a sloped single-path.^70^ That is, the singlet transition (with fewer backward occurrences) takes place around a minimum of the upper state surface with multiple preferred relaxation directions on the lower surface (which should both promote funneling), whereas the triplet transition (with more backward occurrences) takes place on a slope of the upper state with a single downwards pathway in the lower state (which may both inhibit funneling). The tendency for backward transfer of population, in general, would likely be reduced in a solvent by rapid dissipation of kinetic energy and thereby not be directly relatable to previous experiments.^18,20–22^

In contrast to the energies, the similarities in excitation character cannot at all be captured within a Franck–Condon picture. The effect is instead most clearly demonstrated by how intersystem crossing (ISC) is in the current case dominated by S_1_ to T_2_ transfer, which is El-Sayed forbidden as the two states share the same nominal electronic configuration.^71,72^ The ISC must, therefore, be driven by symmetry-breaking in the structural dynamics, where the already noted similarity in both energy and localization of the *n_S_* and *π_S_* orbitals then allows for significant mixing of the electronic characters. Based on a single point calculation of the MECP geometry between the S_1_ and T_2_ states (which represents well the hopping geometries as shown in Sec. III C), we find as leading terms of the configuration expansions for the states ${\text{S}}_{1}=0.69(n_{S},\pi^{*})+0.58(\pi_{S},\pi^{*})$ and ${\text{T}}_{2}=0.73(n_{S},\pi^{*})-0.58(\pi_{S},\pi^{*})$. The two states are, thereby, essentially orthogonal in their spatial wave functions, similarly as expected for the “El-Sayed allowed” transitions, but consist, in contrast, of heavily mixed contributions from in-plane and out-of-plane excitation characters. Similarly, for a sampling over five randomly selected hop-inducing geometries (as introduced in the Sec. III C) for the same transition, the weight of the dominant configuration has a mean (standard deviation) of 0.63 (±0.13) and 0.81 (±0.14) for the S_1_ and T_2_ states, respectively. It can, for these reasons, also be assumed that all the electronic states, as they all nominally involve excitations between the same orbitals, similarly carry a mixed character throughout parts of the non-adiabatic dynamics.

**FIG. 4.**
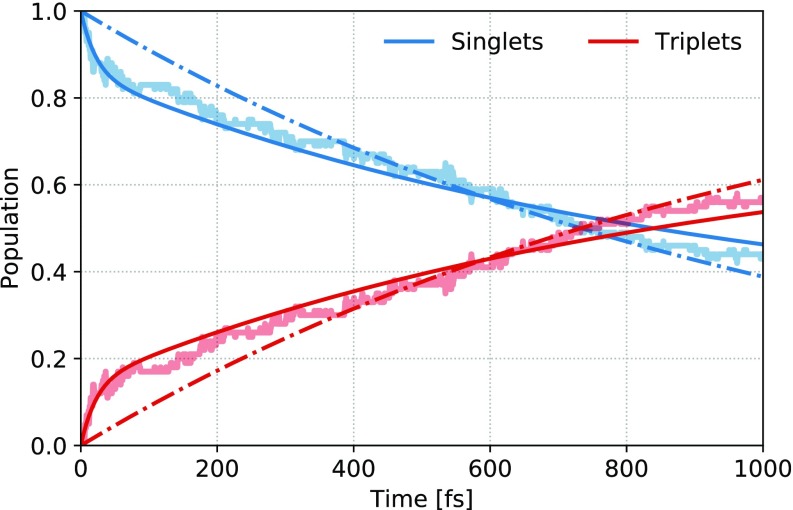
Singlet and triplet state populations (summed over individual MCH states, thick shaded lines) compared to the full kinetic model fit (thin full lines, see supplementary material Sec. S4 for further details) and a simple exponential fit with a lifetime of *τ* = 1058 fs ∼1 ps of the singlet manifold (dashed lines).

While the kinetics of the individual electronic states appear to be difficult to model, a simple trend emerges when they are summed together according to their spin multiplicities. The decay of the singlet manifold population, as shown in Fig. 4, can be almost as well described with a single exponential fit as with the full kinetic model. The simplified fit allows us to better quantify the triplet state population transfer, with the rate constant that translates into an ∼1 ps lifetime of the singlet manifold. The result is consistent with x-ray measurements in solutions, where T_1_ appears to be dominantly populated within the time resolution of ∼20 ps.^21,22^ The trend of the efficient triplet population upon thionation observed for a number of compounds,^32^ thereby, appears to hold also with the active involvement of more electronic states in the reaction pathway. Further extrapolated within the 2-TP system, this trend would explain why the same reaction pathway was also observed for S_4_ excitation at pico- to nanosecond scales,^22^ through similar ultrafast relaxation to the triplet manifold.

### C. Structural dynamics

**FIG. 5.**
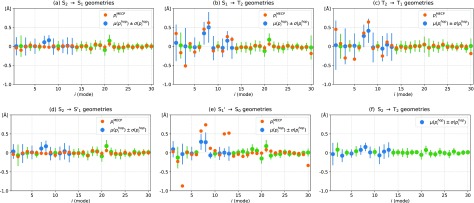
Transition-inducing molecular geometries: comparison of the molecular geometries that precede a surface hop in the SHARC simulations (hop) to MECP geometries. The geometries are expressed as distortions along the normal mode coordinates of the electronic ground state, with each data point as the projection (12 × 3 dimensional scalar product) $p_{i}=\langle \Delta {\vec{r}}_{\mathrm{geo}}\cdot \Delta {\hat{r}}_{NM,i}\rangle$ between the molecular geometry distortion $\Delta {\vec{r}}_{\mathrm{geo}}={\vec{r}}_{\mathrm{geo}}-{\vec{r}}_{\mathrm{Franck}-\mathrm{Condon}}$ and the normalized normal mode displacement vector $\Delta {\hat{r}}_{NM,i}$ of mode *i*. Green dots mark in-plane modes and blue dots out-of-plane modes. The out-of-plane modes were, for each geometry, if needed, aligned to a positive phase of mode 8 by a mirroring through the molecular plane, to facilitate comparison to the MECPs.

**FIG. 6.**
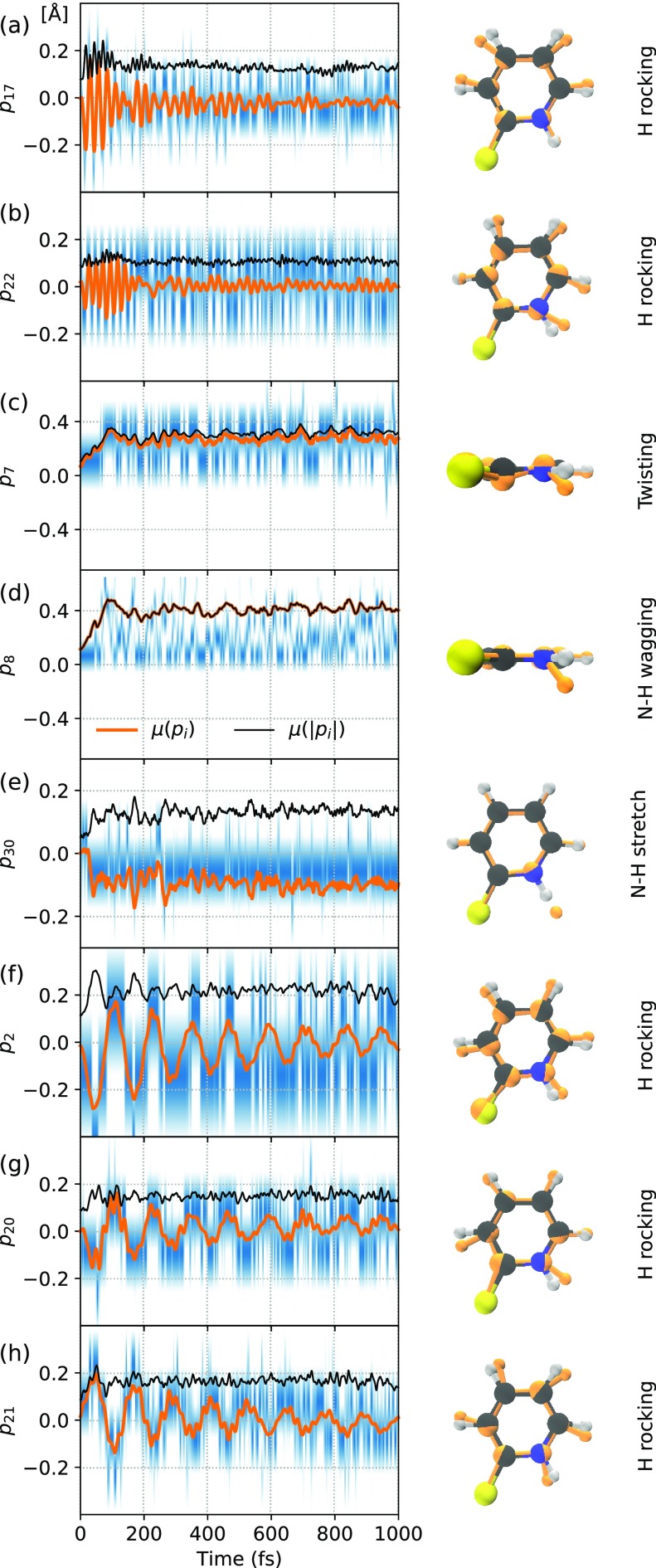
Time evolution of selected normal mode coordinate projections *p_i_* (as described in Fig. 5). The blue colored heat plots represent the binned trajectory density for a given time. Orange lines mark the ensemble average and black lines the ensemble average of the absolute values. The normal mode coordinates are visualized to the right, as an orange distortion of the optimized geometry.

With an overview of the population transfer kinetics, we now turn to the structural dynamics. To rationalize how the structural distortions drive the electronic transitions, we first consider the “hop-inducing” geometries. Figure 5 shows, for each forward transition within the main and the minority pathways, the mean value *μ* and standard deviation *σ* of all geometries that precede the corresponding surface hops. The backward transitions, as shown in supplementary material Fig. S6, show no appreciable differences. The geometries are expressed in normal mode coordinates, some of which are visualized in Fig. 6, while the rest can be found in supplementary material Figs. S7–S9. The data are further compared with pair-wise MECP geometries, visualized in supplementary material Fig. S5.

Starting with the main pathway, the initial internal conversion (IC) from S_2_ to S_1_ occurs through minimal structural distortion. This can be rationalized by their small energy gap already in the Franck–Condon region and is consistent with the ultrafast population transfer from S_2_ to S_1_ (see Fig. 3). In contrast, the S_1_ to T_2_ ISC is driven by strong distortions in almost all out-of-plane modes (blue dots in Fig. 5). This is, as previously discussed, necessary to enable the spin-flip through mixing of electronic state characters, which would, otherwise, be highly inefficient between a singlet and triplet state of the same configuration.^71,72^ A large number of apparent transitions within the triplet manifold then proceed through similar out-of-plane motion. Yet, considering the minuscule energy separation of the T_1_ and T_2_ states already in the Franck–Condon region and the character mixing induced by the symmetry breaking, it is likely that most of these reflect a rapid and erratic energy re-ordering rather than transitions between well distinguishable states.

The first minority pathway results from S_2_ to T_2_ ISC through small structural distortions. It, thereby, represents the “El-Sayed efficient”^71,72^ $\left(\pi ,\pi^{*}\right)$ to $\left(n,\pi^{*}\right)$ ISC, in contrast to the El-Sayed forbidden $\left(n,\pi^{*}\right)$ to $\left(n,\pi^{*}\right)$ ISC of the main pathway. In further contrast, this ISC channel also competes with the S_2_ to S_1_ IC, as S_2_ is not the lowest state within the manifold of excited singlets. An appreciable ISC yield already from the S_2_ state, thereby, appears to directly reflect the increased ISC rate by the heavy atom effect of the sulfur. Still, a significant majority of ISC occur after relaxation within the singlet manifold to S_1_, as traditionally expected.

The second minority pathway results from S_1_ to S_0_ IC. To separate this from the main pathway that instead proceeds through ISC, we denote the minority pathway trajectories with an alternative species ${\text{S}}_{1}^{\prime }$. While the split between S_1_ and ${\text{S}}_{1}^{\prime }$ is for fitting purposes implemented in the decay from S_2_, we deduce that it actually occurs in the subsequent dynamics on the S_1_ surface as presented in Fig. 2. This as S_1_ and ${\text{S}}_{1}^{\prime }$ are reached through highly comparable geometries [compare Figs. 5(a) and 5(d)] but decays through more distinguishable geometries [compare Figs. 5(b) and 5(e)] on different timescales: ${\text{S}}_{1}^{\prime }$ to S_0_ occurs on average after 114 fs, whereas S_1_ to T_2_ occurs on average after 369 fs. The splitting is, therefore, likely to be (partially) induced by the bifurcating character of the S_2_–S_1_ MECI, mentioned in Sec. III B.

The dynamical picture based on the hop-inducing geometries can be compared with a static perspective provided by the MECP geometries also presented in Fig. 5. We note that the MECP data for out-of-plane modes, due to the planar symmetry that makes their sign arbitrary, must be compared to the total distortions enabled by both the mean values and standard deviations of the hop statistics. The exception to this is mode 8 and any other mode coherently phased to it, to which the signs were aligned to facilitate comparison. For the main pathway, we mainly note that the results appear to be in overall acceptable agreement, i.e., the transitions appear to, in most cases, occur “close” to the MECP, which thereby validates the argumentation about electronic state characters for the associated ICs and ISC invoked in the last section, based on single point calculations at the MECPs (MECIs). Regrettably, the S_2_ to T_2_ MECP of the first minority pathway could not be converged, due to the complications that arise from S_1_ being energetically situated between the two states. Finally, the S_1_ to S_0_ MECI predicts much larger distortion, in several modes, than seen in the dynamics. This can be understood by how the transitions, on average, occur for a potential energy of 1.33 eV above the MECI and results in a 0.69 eV increase in kinetic energy, which shows that the transitions typically occur far from both the MECI and any degeneracy between the two surfaces.

Finally, we consider the structural dynamics also in a fully time-dependent picture. Figure 6 presents the time-evolution of selected normal mode coordinates (i.e., those that display notable changes in amplitude over time and/or clear coherence) over the whole ensemble of trajectories. To gain some measure of the coherence between trajectories, we compare the mean values of the signed and unsigned coordinates. Equal amplitudes (of, e.g., a periodic oscillation or systematic drift) of the two then signal a completely coherent motion, whereas a vanishing average of the signed coordinates oppositely signals a complete dephasing.

Based on the described analysis, the following picture emerges: the dynamics are initially dominated by coherent in-plane H rocking, particularly in modes 17 and 22. As the S_1_ state reaches its peak population and starts to decay, these modes dephase, as signaled by a rapid decrease in amplitude for the signed coordinates, whereas the amplitudes of the unsigned coordinates remain comparable. Concurrently, out-of-plane motion steadily increases over the first 100 fs and thereafter remains comparable throughout the remainder of the simulations. This is primarily seen from N–H centric modes 7 and 8 with unsigned values that drastically increase before flattening out. Due to the arbitrariness in the sign of the out-of-plane modes, however, the signed values appear to be artificially dephased. Within the same time frame, mode 30, which is essentially a pure N–H stretch, decreases notably in amplitude, i.e., the bond contracts from the S_1_ state and on. Finally, an additional number of H rocking modes, in particular, 2, 20, and 21, display coherent motion over the whole time range. A partial dephasing, from the initially high coherence, occurs steadily over time, seen from a larger decrease in the sign than the unsigned coordinates, but with clear oscillations that remain even to the end of the simulations.

### D. Proton transfer

A complete ESPT is not found in any of the currently presented trajectories. Yet, large variations in the bonding structure are still observed, as demonstrated by the maximal $r_{N-H}$ value of 1.41 Å and the minimal $r_{S-H}$ value of 1.99 Å, as compared to their means (with standard deviations) of 1.03 (±0.06) Å and 2.78 (±0.22) Å. While limitations in the statistics still cannot be excluded as a possible cause, this suggests that the absence of ESPT, in the current work, might instead be the result of limitations in the computational model. Such limitations could include (but not necessarily be limited to) exclusion of solvent molecules (which might, in fact, mediate both the proton detachment and reattachment in the measurements), insufficient flexibility in the electronic structure method, or limitations imposed by the classical dynamics. Nevertheless, the observed electronic and structural dynamics still allow us to speculate about which aspects of the photo-dynamics could promote ESPT.

Starting from the Franck–Condon picture, it is unclear why any of the electronic excitations should promote ESPT, as they all appear to transfer electronic density from the sulfur to the nitrogen site, which should increase the basicity of the former and acidity of the latter. A Mulliken analysis, in fact, shows that the initial −0.46 net charge of the sulfur site increases notably to −0.04 (S_1_), −0.25 (S_2_), −0.19 (T_1_), and −0.09 (T_2_) upon excitation, with smaller effects for the nitrogen site. This clearly suggests that the ESPT must instead result from dynamical or external factors.

In terms of the structural dynamics that occur on short timescales (∼100 fs), mode 22 is most likely to promote ESPT as it clearly rocks the N–H and C–S bonds with respect to each other, which could provide a channel for ultrafast intra-molecular ESPT before the dephasing of modes 17 and 22 sets in. Such a mechanism would be consistent with the weak signature of N–H cleavage previously observed using femtosecond RIXS.^20^ It could, however, prove to be difficult to satisfactorily investigate using computational models, as it may require both a quantum description of the dynamics (to, for instance, account for tunneling of the proton through a classically forbidden barrier) and significant statistics to either confirm or reject. Modes 2, 20, and 21 could similarly participate in an intra-molecular transfer as they also affect the S–H distance, but they all exhibit time periods of roughly 150 fs, i.e., comparable to the time point where out-of-plane motion instead sets in to dominate.

In less than 100 fs, the N–H bond on average contracts, which should inhibit its cleavage. ESPT on longer timescales is, therefore, likely to be mediated by external factors, which strongly points toward interactions with one or several solvent molecules. The bond contraction onsets together with the strong out-of-plane motions, in particular, N–H centered modes 7 and 8, which, in a solution environment, could strongly affect the proton's coordination with the solvent. In our previously mentioned Born–Oppenheimer molecular dynamics study,^23^ we further found both that the compound exhibits strong hydrogen bonding with aqueous solutions and that the nitrogen site coordinated water molecule of the anion species 2-TP^−^ interchanged through a rotation that realigned the molecular plane with respect to the solvent. A similar mechanism, i.e., solvent re-coordination through out-of-plane motion, could, therefore, also be responsible for solvent-mediated ESPT at timescales above 100 fs. This would be consistent with pico- to nanosecond NEXAFS measurements, which find the ESPT to be a rare event, presumably mediated by large fluctuations that concurrently result in T_1_ to S_0_ relaxation.

Based on currently presented and recent insights,^23,24^ we finally want to point out two clear ways that time-resolved spectroscopy could be constructively employed to shed further light on the ESPT of 2-TP: (i) femtosecond timescale measurements, with a probe capable of resolving either electronic states or vibrational modes, would be particularly useful to evaluate speculations about the ESPT mechanism and pathway from current and previous work.^18–20^ (ii) Gas phase measurements on any timescale from femto- to pico- to nanoseconds, with a probe sensitive to the nitrogen and/or sulfur protonation, could conclusively determine whether a solvent is necessary to mediate the ESPT. Insights derived from such experiments could, possibly, fill all the significant gaps in the current understanding of the pathway to fully “solve” the ESPT process of 2-TP.

## IV. CONCLUSIONS

Non-adiabatic dynamics of 2-TP have been simulated by surface hopping simulations including spin–orbit induced transitions, modeling the UV-induced excited-state dynamics probed in multiple previous time-resolved spectroscopic measurements.^18,20–22^

An ultrafast S_2_ to S_1_ population transfer, mediated by coherent in-plane vibrations, is followed by complex population kinetics that appreciably populates all the energetically available states through multiple pathways. The complexity results from out-of-plane structural dynamics that onset in the S_1_ state and significantly mixes the excitation character of the electronic states, which, in particular, drives ISC through the El-Sayed forbidden ${\text{S}}_{1}(n,\;\pi^{*}$) to ${\text{T}}_{2}(n,\;\pi^{*}$) channel. The transfer of the population from singlets to triplets, once summed over the individual states, can nevertheless be well described as a simple exponential decay with a lifetime of ∼1 ps. This follows the trend of efficient triplet population observed for multiple similar thio-substituted compounds and offers rationalization for why the same excited-stated dynamics have been previously observed for both S_2_ and S_4_ excitation of the system, on picosecond scales and longer.^22^

While no ESPT was observed in the current work, two aspects of the structural dynamics are suggested as possible mediators for it in previously reported experiments: initial coherent in-plane motion for ultrafast (∼100 fs) intra-molecular transfer and out-of-plane motion for solvent-mediated transfer at longer timescales. With this in mind, we suggest two ways in which time-resolved spectroscopy could be employed to address important remaining questions about the ESPT pathway.

## SUPPLEMENTARY MATERIAL

See the supplementary material for comparison to dynamics performed with a larger basis set, estimation of state energy splittings in the dynamics, comparison of electronic state populations in other state representations, comparison to other kinetic model fits, visualization of the MECP geometries, visualization of hop-inducing geometries for backward transitions, visualization of normal modes of the electronic ground state, and example input files for the OpenMolcas and SHARC computations.
